# The Relationship of NADPH Oxidases and Heme Peroxidases: Fallin' in and Out

**DOI:** 10.3389/fimmu.2019.00394

**Published:** 2019-03-05

**Authors:** Gábor Sirokmány, Miklós Geiszt

**Affiliations:** ^1^Department of Physiology, Faculty of Medicine, Semmelweis University, Budapest, Hungary; ^2^“Momentum” Peroxidase Enzyme Research Group of the Semmelweis University and the Hungarian Academy of Sciences, Budapest, Hungary

**Keywords:** heme peroxidase, NADPH oxidase, hydrogen peroxide, reactive oxygen species, peroxidasin

## Abstract

Peroxidase enzymes can oxidize a multitude of substrates in diverse biological processes. According to the latest phylogenetic analysis, there are four major heme peroxidase superfamilies. In this review, we focus on certain members of the cyclooxygenase-peroxidase superfamily (also labeled as animal heme peroxidases) and their connection to specific NADPH oxidase enzymes which provide H_2_O_2_ for the one- and two-electron oxidation of various peroxidase substrates. The family of NADPH oxidases is a group of enzymes dedicated to the production of superoxide and hydrogen peroxide. There is a handful of known and important physiological functions where one of the seven known human NADPH oxidases plays an essential role. In most of these functions NADPH oxidases provide H_2_O_2_ for specific heme peroxidases and the concerted action of the two enzymes is indispensable for the accomplishment of the biological function. We discuss human and other metazoan examples of such cooperation between oxidases and peroxidases and analyze the biological importance of their functional interaction. We also review those oxidases and peroxidases where this kind of partnership has not been identified yet.

## Introduction

Heme peroxidases comprise a large number of heme-containing proteins. Families of these enzymes display distinct structural and biochemical properties and play a role in highly specialized biological processes. The numerous members of each of these families are expressed in all different kingdoms of life. Therefore, it had been recently suggested that the denomination of heme peroxidases should happen according to their characteristic enzymatic activities and structural properties instead of their animal, plant or fungal origin (1). The unique feature of the peroxidase-cyclooxygenase superfamily is the presence of a post-translationally modified heme group which is covalently linked to the peroxidase protein via two covalent bonds (2). Myeloperoxidase (MPO) is unique in this superfamily because of having three covalent linkages to the heme group.

The activity of these peroxidases results in oxidation of one-electron donors into the corresponding radical (AH in Reaction 1) or oxidation of halides or pseudohalides (two-electron donors) into hypohalous acids (HOX in Reaction 2) (3).

$$\begin{array}{l} \text{Reaction}1:{\text{H}}_{2}O_{2}+2{\text{AH}}_{2}\to 2{\text{H}}_{2}O+\;2\cdot \text{AH}\\ \text{Reaction2}:{\text{H}}_{2}O_{2}+{\text{H}}^{+}+{\text{X}}^{-}\to {\text{H}}_{2}\text{O}+\;\text{HOX} \end{array}$$

H_2_O_2_ is not only required for the generation of oxidants but it seems that it is also necessary to the autocatalytic activation of heme peroxidases during which process the heme-protein crosslink is reinforced (4).

Numerous biochemical processes can produce reactive oxygen species including hydrogen peroxide. The mitochondrial respiratory chain, several metabolic pathways, xanthine oxidase, monoamine oxidases, and the NADPH oxidases are all possible sources of H_2_O_2_ (5–7).

There are seven members of the Nox/Duox family of NADPH oxidases encoded in the human genome. In other species the number of NADPH oxidase homologs can vary greatly with only two isoforms present in *Caenorhabditis elegans*, five members in zebrafish, six in mouse and rat to name a few examples (8). The NADPH oxidases show important differences in tissue expression pattern and activation mechanism. Nox1, Nox2, Nox3, and Nox4 all require the membrane-bound p22^phox^ protein to be able to produce ROS. Nox1, Nox2, and Nox3 also require different cytosolic factors to become active (9). However, Nox4 does not rely on cytosolic factors but is continuously active. Nox5, Duox1, and Duox2 are independent of p22^phox^ and are primarily activated by intracellular Ca^++^-signals. Interestingly, Duox proteins can be also classified as heme peroxidases although their peroxidase domain lacks a few critical amino acids that are required for the enzymatic activity of their N-terminal peroxidase domain (10). Dual oxidase (Duox) proteins also require the activity of maturation factors DuoxA1 or DuoxA2 for proper folding and membrane targeting (11).

Hydrogen peroxide is not always the primary oxidant product of NADPH oxidases. Nox2 for example generates mainly superoxide anion, which can be further converted into H_2_O_2_ in a dismutation reaction enhanced by superoxide dismutase (SOD) enzymes (12). SOD catalyzes the disproportionation of the free radical superoxide anion resulting in the generation of molecular oxygen and hydrogen peroxide (see in Reaction 3) (13).

$$\text{Reaction}3:{2\text{O}}_{2}^{-}+2{\text{H}}_{3}{\text{O}}^{+}\to {\text{O}}_{2}+H_{2}O_{2}+\;2{\text{H}}_{2}\text{O}$$

Certain members of the cyclooxygenase-peroxidase family rely specifically on hydrogen peroxide generated by an NADPH-oxidase. In these specific cases, the absence of the corresponding NADPH-oxidase cannot be supplemented by any other H_2_O_2_ sources. This closely intertwined mode of action suggests evolutionarily conserved cooperation between these heme peroxidases and NADPH-oxidases. Our aim was to collect and analyze all known examples of such coactions.

The natural beauty of these peroxidase-oxidase concurrences is literally highlighted by a chemiluminescent light emitted during intense activation of the phagocyte myeloperoxidase or the fertilized sea urchin egg's ovoperoxidase (14, 15).

## Leukocyte Nox2/Myeloperoxidase System

The prodigious increase in oxygen consumption of phagocytosing leukocytes was already described in 1933 by C.W. Baldridge and R.W. Gerard (16). During the following decades, it became clear that this oxygen consumption was not dependent on mitochondrial respiration but was necessary for the production of reactive oxygen species by a complex, multi-protein system that comprised of membrane-bound and cytosolic factors. Nox2 (formerly known as gp91^phox^) contains 6 transmembrane helices and forms a membrane-bound complex with p22^phox^. Whereas, there are 4 cytosolic factors that *in vivo* are all necessary for a fully activated oxidase complex: p67^phox^, p47^phox^, p40^phox^, and the small GTPase Rac1 or Rac2 (9, 17). These cytosolic components are all able to rapidly translocate to the gp91^phox^-p22^phox^ complex upon activation of the phagocyte. The gp91^phox^-p22^phox^ complex is stored in the peroxidase negative subsets of the neutrophil granulocytes' granules which—upon activation—fuse with the phagosomal or plasma membrane. Compared to other Nox isoforms, the ROS producing capacity of the activated Nox2 system seems to be extremely high (18, 19) and this might ensure the potent antimicrobial function of this isoform.

Another line of research elucidated the biochemical activity of myeloperoxidase that was present in large quantities (5% of the total dry cell weight) in phagocytes and was able to turn H_2_O_2_ into microbicidal hypohalides like HOCl (20). Myeloperoxidase is stored mainly in the matrix of azurophilic granules of neutrophil granulocytes in a mature, dimeric form. The dimerization does not seem to affect the enzymatic activity of MPO but it is rather important for the stability and storage of the enzyme (21). In activated leukocytes, the granules can be released into the lumen of the forming phagosome or into the extracellular space around phagocytes (22, 23). The translocation of myeloperoxidase and Nox2 oxidase (cytochrome_b558_ complex of Nox2 and p22^phox^) from cytoplasmic granules and vesicles into the phagosomal lumen was demonstrated by different approaches including studies based on subcellular fractionation, fluorescent and electronmicroscopic analysis (24–27).

The above regulatory mechanisms, multi-component assembly, and compartmentalization ensure that in non-stimulated cells there is practically no hypohalide production. This can prevent that aggressive antimicrobial reactive oxygen products cause random tissue destruction in the host.

Notably, human phagocytes express about an order of magnitude higher amounts of MPO than mouse leukocytes which limit the interpretation of mouse data. However, to circumvent this problem mouse models expressing human MPO have been established (28, 29). These models became important also to study the role of MPO in the pathomechanism of atherosclerotic lesion formation, as human atherosclerotic lesions do contain MPO and its peroxidation products whereas mouse MPO is hardly detectable in macrophages of atherosclerotic plaques. The expression of human MPO in mouse macrophages was unanimously associated with increased atherosclerotic lesions in different studies. However, other functional changes, like plasma lipoprotein and cholesterol levels showed more conflicting results, which might be explained by different transgenic systems used in different studies [i.e., bone marrow transplanted transgenic macrophages (28) or overall expression of the MPO transgene (29, 30)].

Similarly to the numerous other antimicrobial effector functions of phagocytes, dysregulation of superoxide production and myeloperoxidase activity can also contribute to the development of autoimmune diseases. Both the over activation or impairment of these processes can promote tissue damage associated with autoimmune conditions (31).

Furthermore, the lack of either Nox2 or MPO does result in immune deficiency disorder with substantially different characteristics and severity. In the absence of Nox2—or other components of the active phagocytic oxidase complex—a disease called chronic granulomatous disease (CGD) develops (32). CGD patients have largely increased susceptibility toward both bacterial and fungal infections. In contrast, many patients with loss of function mutations of MPO might have no obvious clinical symptoms or show an increased predisposition only toward fungal infections (caused mainly by *Candida albicans*) (33, 34). This phenotypic discrepancy might be explained in different ways. First, it is possible that the superoxide produced by Nox2 and/or its derivatives exert direct antimicrobial effects even without being converted into hypochlorous acid. Second, based on observations showing altered membrane potential changes and Ca^++^-signals in CGD neutrophils it is possible to presume that altered intracellular ion concentrations might hamper also several other antimicrobial effector functions of these cells (35, 36).

## Eosinophil Nox2/Eosinophil Peroxidase System

Although the eosinophil peroxidase (EPO) and myeloperoxidase are highly homologous at the amino acid level their biochemical properties and biological role differ significantly. EPO binds its prosthetic group only via two covalent links and its spectral properties are more similar to that of LPO and TPO (2). Eosinophil granulocytes also express components of the Nox2 based superoxide-generating molecular machinery which provides H_2_O_2_ for EPO. Unlike MPO, EPO is not able to oxidize Cl^−^, but it uses mainly Br^−^ and SCN^−^ to generate hypobromous acid and hypothiocyanous acid, respectively. Detection of protein bromotyrosination can be used as a marker of EPO mediated protein oxidation (37).

Eosinophil granulocytes exert their antimicrobial and antiparasitic activities extracellularly and EPO is also a secreted protein. Eosinophils also play a special role in the pathologies of allergic inflammatory diseases (38–40). Despite the essential host defense and inflammatory role of eosinophil granulocytes the lack of eosinophil peroxidase activity does not manifest in any obvious phenotype in humans and the diagnosis of EPO deficiency is usually an accidental clinical finding (41). In contrast, in allergic diseases, accumulation and hyperactivity of eosinophil granulocytes are associated with overproduction of oxidative substances which contributes to the pathology of these conditions. An especially interesting pathomechanism is the activation of endothelium-derived tissue factor by hypothiocyanous acid with the consequentially increased risk of thrombotic complications (42, 43).

## Duox2 and Thyroid Peroxidase in Thyroid Hormone Synthesis

The thyroid peroxidase (TPO) catalyzes the iodination of tyrosine residues of thyroglobulin (44, 45). This peroxidase has got a unique transmembrane domain through which it is located in the apical membrane of thyrocytes. Its heme-containing catalytic site faces the thyroglobulin containing follicular lumen. To produce active iodine radicals the thyrocytes take up iodide ion through the basolateral Na^+^/I^−^ symporter and transport it to the lumen through pendrin or through other apically located anion transporters (46). H_2_O_2_ is produced by dual oxidase 2 (Duox2), an other apically located transmembrane NADPH oxidase enzyme. The pharmacological inhibition or loss of function mutations of I^−^ transporters, TPO or Duox2, and DuoxA2 all lead to insufficient thyroid hormone synthesis—i.e., hypothyreosis.

As we have no detailed structural insight into the molecular vicinity of the luminal site of the thyrocyte, it is difficult to explain how the produced, highly reactive iodine radicals can react selectively with tyrosine side chains of thyroglobulin without eliciting oxidative damage of other extracellular proteins. Co-immunoprecipitation studies revealed a molecular interaction between TPO and Duox2 in the membrane fractions of isolated thyroid tissue lysates and of transfected COS-7 cells as well (47). This close association can at least explain how leakage of H_2_O_2_ can be prevented.

In contrast to the Nox2/MPO system, the thyroid hormone synthesis is a rather continuous, steady process. Accordingly, the oxidase and peroxidase components are located in the same subcellular compartment. Interestingly, the thyroid expresses two dual oxidases, Duox1 and Duox2 (48), but the absence of Duox1 is not associated with hypothyreosis (49). Therefore, the exact function of Duox1 in the thyroid is still unknown. It is also quite enigmatic why the highly homologous Duox1 cannot compensate for the lack of Duox2 in the hormone synthesis process. One explanation might be that Duox1 localizes to another microdomain of the apical membrane. However, the lack of Duox1 specific antibodies that work in immunohistochemistry applications makes it difficult to prove this idea (50).

## Duox and Lactoperoxidase in Exocrine Glands and on Mucosal Surfaces

Lactoperoxidase (LPO) has long been recognized as an antimicrobial enzyme present in various exocrine secretions like milk, saliva, and tear. In 2003 a detailed *in situ* hybridization study identified Duox2 expression in major salivary ducts and on rectal epithelial cells and Duox1 expression in airway epithelial cells (58).

LPO can utilize I^−^ or SCN^−^ as substrates and the sodium/iodide transporter (NIS) plays an important role in transporting these anions through the epithelial cells. In the salivary glands LPO was expressed deep in the serous acini, NIS in the intercalated ducts and Duox2 in final ducts (58). This pattern of expression could ensure that the microbicide hypothiocyanous acid is formed only at later stages of secretion, just before entering the oral cavity.

In the airways, thiocyanate might be transported onto the epithelial surface via the cystic fibrosis transmembrane regulator CFTR (58–62). Decreased transport activity in cystic fibrosis patients might reduce the LPO mediated antimicrobial effects which might contribute to the high rate of pulmonary infections. Importantly microbes seem to be much more susceptible to HOSCN than mammalian cells probably because mammalian epithelial cells express high molecular weight thioredoxin reductase (TrxR) that can readily turn over HOSCN. In contrast, bacterial TrxR is strongly inhibited by HOSCN (62). This makes the Duox-LPO-HOSCN system much more adequate for continuous mucosal host defense functions than the more cytotoxic Nox2-MPO-HOCl system (63, 64).

## *C. elegans* Dual Oxidase 1 (BLI-3) and Heme Peroxidases

The *Caenorhabditis elegans* NADPH oxidase, BLI-3 was described to be expressed in the hypodermal cells of *C. elegans* underlying the cuticle layer. The hypodermal cells play an essential role in the repeated synthesis of the cuticle (molting) during consecutive larval stages of the worm. RNAi knockdown of BLI-3 resulted in severe cuticle abnormalities. Di- and trityrosine crosslinks between cuticular collagen molecules were found to be significantly reduced in BLI-3 RNAi worms. The same study that described this phenotype also proposed that the BLI-3 peroxidase domain was responsible for the tyrosine crosslinks between cuticular collagens (65). However, later analysis revealed that the BLI-3 peroxidase domain lacks critical amino acids that are important for heme binding which makes its peroxidase activity doubtful. Accordingly, a reverse genetics RNAi screen approach identified the hypodermally expressed MLT-7 peroxidase that was responsible for collagen crosslinking (55). MLT-7 expression showed a cyclic pattern according to molting stages and its knockdown showed very similar phenotypes to BLI-3 knockdowns. It has been supposed that the enzymatically inactive BLI-3 peroxidase domain might function as a docking site for MLT-7 peroxidase domain thereby providing a spatial control of the peroxidase activity (55).

Another hypodermally localized heme peroxidase—SKPO-1—was also discovered by RNAi screening that was found to be important in maintaining normal cuticle phenotype. SKPO-1 was also claimed to play a role—along with BLI-3—in host defense against pathogenic bacteria (56, 66).

## Ovoperoxidase and Urchin Dual Oxidase 1 in the Sea Urchin Fertilization Membrane

In the sea urchin (*Strongylocentrotus purpuratus*) the fertilization of the egg elicits plasma membrane depolarization, cytosolic Ca^++^-signal and a cortical reaction which involves degranulation of vesicles located below the egg's membrane surface (67). This results in the formation of a stiff, insoluble fertilization envelope (FE) which prevents the entry of other sperms (53). The active pool of the sea urchin oxidase is located in the egg's plasma membrane whereas the ovoperoxidase is tethered to the forming FE by a protein called proteoliaisin (54, 68). Bennett M. Shapiro and his coworkers gave a detailed description of this envelope formation, they isolated and characterized the ovoperoxidase enzyme that is released from the granules and is responsible for the crosslinking of protein tyrosyl residues in this hardened membrane. Another important feature of the secreted peroxidase is its spermicidal activity which means an additional defense mechanism against polyspermy. Although the source of H_2_O_2_ for these peroxidase mediated reactions were already addressed in the 1977 PNAS paper, the molecular identification of the urchin dual oxidase (Udx1) was accomplished almost three decades later (54). This process is also a prime example of how compartmentalization and inducible translocation can ensure a swift, robust, but tightly controlled oxidative burst and peroxidase activation.

Although it is challenging to find quantitatively comparable data about the ROS production of different NADPH oxidase systems, it seems that the respiratory burst in the sea urchin egg results in a H_2_O_2_ concentration of about 60 nM in the perivitelline space whereas in the phagosome of activated human neutrophil granulocytes the peroxide concentration is estimated to be in the micromolar range (54, 69). However, in the biological context of egg fertilization, this relatively lower rate of ROS production can still amply support the ovoperoxidase function.

## Nox5 and Heme Peroxidase 2 in Mosquito Antiplasmodial Immunity

A unique example has been identified in the midgut cells of Anopheles gambiae where the HPX2 heme peroxidase—in concert with Nox5 and nitric oxide synthase (NOS)—generates reactive nitrogen species (RNS) resulting in increased protein nitration. This process renders the Plasmodium ookinetes more susceptible to the Anopheles complement system which is the final effector mechanism against invading parasites (57) (see Table 1).

**Table 1:** An overview of the peroxidase-oxidase co-operations discussed in detail in this paper.

**Peroxidase**	**Oxidase**	**Location**	**Function**	**Related anomaly or disease in the absence of oxidase/peroxidase function**
MPO	Nox2	Neutrophil granulocytes, macrophages, peritoneal B lymphocytes	Production of antimicrobial hypochloric acid	Nox2: chronic granulomatous disease (CGD) (32) MPO: increased susceptibility to fungal infections (34)
EPO	Nox2	Eosinophil granulocytes	Production of antimicrobial hypobromous and hypothiocyanous acid	no evidence of disease (51)
TPO	Duox2	Thyroid gland	Oxidation of iodide ion during thyroid hormone synthesis	Duox2 or TPO: congenital hypothyreosis (52) (48) (44) (45)
LPO	Duox1, Duox2	Exocrin glands, mucosal surfaces	Production of antimicrobial hypothiocyanate and hypoiodide	?
Ovoperoxidase	Udx1	Plasmamembrane, subcortical granules, fertilization envelope	Crosslinking and subsequent hardening of matrix molecules in the fertilization membrane	Ovoperoxidase or Udx1: increased probability of polyspermy (53) (54)
MLT-7	BLI-3 (Ce-Duox1)	Hypodermis	Crosslinking of cuticle matrix molecules	BLI-3 or MLT7: developmental arrest, cuticle abnormalities (55)
SKPO-1	BLI-3 (Ce-Duox1)	Hypodermis	Maintaining normal cuticle, host defense	BLI-3 or SKPO-1: developmental arrest, susceptibility to Enterococcus infection (56)
HPX2	NOX5	Plasmodium infected Anopheles midgut epithelial cells	Nitration of Plasmodium ookinetes	Nox5 or HPX2: susceptibility to Plasmodium invasion (57)

The same research group presented another mechanism in the Anopheles midgut where a Duox homolog cooperates with a secreted immunomodulatory epithelial peroxidase (IMPer) to form a dityrosine crosslinked matrix on the luminal surface. This matrix layer is supposed to separate the luminal microbiota and the epithelial immune system thereby subduing the immune activation and potential epithelial damage. On the other hand, Plasmodium parasites could proliferate more rapidly in the midgut lumen under these conditions (70).

## Nox1, Nox3, and Nox4 Function Without Known Peroxidase Partners

These three members of the NADPH oxidase family have not been linked to heme peroxidases in any known biological process yet. Nox1 is mainly expressed in the distal parts of the gastrointestinal tract, showing low expression levels in the ileum, and more robust levels in the colon epithelium (19, 71). In a glutathione peroxidase deficient animal model of spontaneous ileocolitis, it was shown to be involved in the pathogenesis of inflammatory bowel diseases (72). This and other potential physiological functions of Nox1 are reviewed in detail elsewhere (73). However, cooperation with a known heme peroxidase has not been described in the divergent actions of Nox1.

Nox3 is uniquely expressed in the inner ear (74). Although there are no published data about any connection to heme peroxidases in this organ, it is interesting to note that—according to the publicly available NCBI Unigene database—myeloperoxidase has got a surprisingly high expression level in the mouse inner ear. Whether there is any functional link between MPO and Nox3 in the inner ear remains to be investigated.

The Nox4 expression is more ubiquitous with the highest levels found in the kidney. Uniquely this oxidase is constitutively active. The exact intracellular localization of Nox4 is still dubious, however many independent literature data points toward the endoplasmic reticulum where Nox4 might contribute to the oxidative milieu of the ER (75–77). It would be a challenging task to pinpoint any specific Nox4-heme peroxidase functional interaction within this compartment.

## Peroxidasin Without Known Oxidase Partner in Collagen IV Crosslinking

Peroxidasin (Pxdn) has been described as the specific enzyme catalyzing the formation of sulfilimine covalent crosslink of C-terminal NC1 domains between collagen IV protomers (78). This unique, evolutionarily conserved chemical bond might significantly affect the mechanical and biochemical properties of collagen IV containing extracellular matrix structures. However, using various NADPH-oxidase deficient mouse models, it has been recently shown *in vivo* that NADPH oxidases most probably do not provide H_2_O_2_ for this reaction. P22^phox^ mutant, Nox4 deficient, Duox1 knockout, and Duoxa double knockout animals were all equally capable to crosslink NC1 domains as wild-type control animals (79). Lysyl oxidases which are also involved in collagen IV assembly were also ruled out as possible ROS sources (78). Therefore, the exact molecular identity of this reactions' ROS source is still unknown. It is also a puzzling question how the Pxdn mediated reaction is restricted only to the collagen IV NC1 amino acids and how it is ensured that the highly reactive HOBr is not attacking numerous neighboring matrix molecules (80). Identification of a specific ROS source might help explain the spatiotemporal control of redox modification.

## Conclusion

Our review describes the functional cooperation between members of the peroxidase-cyclooxygenase family and NADPH oxidases in various biological settings. The deeper understanding of these processes might help identify novel biological targets of oxidase and peroxidase products and improve our understanding of how these reactive oxidants can contribute to very specific, sophisticated physiological phenomena, or how they can trigger pathophysiological conditions.

## Author Contributions

All authors listed have made a substantial, direct and intellectual contribution to the work, and approved it for publication.

### Conflict of Interest Statement

The authors declare that the research was conducted in the absence of any commercial or financial relationships that could be construed as a potential conflict of interest.
